# Mr. Davies on Mineral Poisons Entering the Circulation

**Published:** 1813-02

**Authors:** D. H. Davies

**Affiliations:** No. 27, Carburton-street, Fitzroy-square


*To the Editors of the Medical and Physical Journal.*

GENTLEMEN,

IN the last Number but one of your valuable work, I published an account of some children who were poisoned from the effects of Arsenic; I shall now enlarge upon the subject, and extend the observations connected with that occurrence.

In the elucidation of truth, and establishment of facts, numerous obstacles are to be encountered, and various prejudices combated; but the diligent investigator, in my opinion, will more essentially serve the cause of science, and the illustration of truth, by courting in any varied shape and manner the experience and opinions of others, on any controverted and dubious subject, than by endeavoring to sweep away, with despotic influence, the systems and hypotheses of his predecessors. However keen his acumen may be, or however varied and extensive his experience may have proved, yet medical facts lie hid under so many disguises, and are obscured by so many circumstances, that the establishment of any principle in physic ought never to rest in the isolated opinion of any one individual, but in the collected statement of the many. Actuated by this mode of reasoning, I am induced to blend questions with opinions, and to solicit information, at the same time that I offer my individual opinion. The obstinate or misinformed, I should hope, are the only characters, who, having broached opinions, will endeavor to maintain and defend those theories against the better information of their own judgment.

It may be recollected that, in the paper I alluded to, a sentence like the following was inserted, viz. A considerable time must necessarily elapse between mineral poison being received into the stomach, and being taken up into the circulation. A most intelligent friend, on reading this sentence, expressed his disapprobation of its being positively stated so. He observed, that it had not been clearly ascertained to be a fact, that mineral poison was taken up by the circulation, or that it destroyed, by contaminating the general mass of circulating fluid. This is a point, he hinted, that was entirely unsettled and fluctuating, and, therefore, conformable with the tenor of my observations at the commencement of this paper. I would solicit information from those who possess much more enlarged abilities than myself, and who have had opportunities, from experiments of comparative anatomy, of practically corroborating or discovering the fact, as far as such experiments will afford proof; to me, however, at present, it appears almost conclusive, that the morbid effects of mineral poison, especially arsenic, are more apparent in the circulation than any where else, and upon those reasons I build my opinion; first, that, in the few cases that came under my observation, the arterial action observable by the pulse has been materially altered, distended, and increased; that, in the two cases in which I have witnessed blood being taken away, evident marks of inflammation existed, especially in the case which which I alluded to in my first paper, which occurred at Nottingham; and, thirdly, that heat, thirst, and dryness, (concomitants of inflammation,) were symptoms in both these cases. As a further illustration of this opinion, the appearances observable after death may be adduced as strongly presumptive, of mineral poison, especially arsenic, destroying principally by exciting excessive inflammation; and, if reference is made to my former paper, it will be found that, on examining the internal and external coats of the intestines, &c. particularly of one of the children, the most decided marks of violent inflammation existed. This, surely, is another strong presumptive proof that arsenic is taken up by the circulation, and proves fatally deranging to that circulation.

In suggesting my opinions on this part of the subject, I by no means wish it to be understood that I consider the stomach as a viscus free from the inroads of the poison; on the contrary, I conceive that this viscus primarily and most severely suffers, and corrosion of its coats may be the consequence; but I do not conceive that any corrosion, abstractedly considered, can be the exciting cause of almost immediate death. One additional circumstance may still further tend to corroborate the opinion that the sanguiferous system is more the subject of morbid derangement, viz. the *marked* alternation and variation in the arterial action, observable in the case of one of the children; that at times the pulse would hardly be perceptible, and symptoms of the greatest prostration and languor would prevail; the pulse, however, would shortly rise again in strength and fullness, and every symptom of increased arterial action become apparent. Such are the principal arguments that have hitherto biassed my judgment in favor of the opinion I now hold with regard to this subject; how correct and justifiable they are, I will not pretend to say; but I fondly cherish the hope that the question will be minutely canvassed and investigated by men who are qualified for such researches.

It is a question of high importance to ascertain satisfactorily in what way the introduction into the system of arsenic destroys life. It is not a question of a speculative nature only, but one in which the advancement of medical knowledge and the good of mankind are equally blended; and the solution of it is the pivot upon which a rational and approved mode of treatment must turn. When I took the liberty of offering some observations on what appeared to me ought to be the first and most immediate plan of of treatment to be adobted in these unfortunate cases, arising from the exhibition of arsenic, I ventured to recommend the use of the lancet, with a view of counteracting inflammation. This mode of practice being built on the theory I have endeavored to substantiate in the preceding part of this paper, I feel it a duty to urge again the early evacuation of blood. Some physicians have advised the application of leeches, but I must confess, for my own part, I should place *no* reliance on their use. The effect of mineral poison is seldom, I think, local, but constitutional, and therefore arresting the general course of the circulation must have a decided preference over the trifling evacuation from a few superficial vessels. The quantity necessary to be taken away must depend on age, and circumstances of the case.

It may be here asked, if the assertion is correct that a considerable time must necessarily elapse between arsenical poison being received into the stomach, and its being taken up by the circulation; why advise the immediate employment of venesection? In reply, I would observe that, however early a medical practitioner may be called in in these cases, and however expeditious he may be in clearing the contents of the stomach, he can never be certain, even with the additional means of evacuating the bowels, that the whole of the pernicious substances has been brought away; and, as even a small quantity remaining in the system is as much, perhaps, or more, likely to induce violent inflammation, the adoption of the practice recommended, at an early period, is, I think, likely to be eminently serviceable.

Having made these observations, I shall now dismiss the subject for the present, with an assurance that they have been offered rather with a view of soliciting information than with any other object; and the opinions thrown out have naturally resulted from the observation of a few cases that have come under my own cognizance.

I remain, Gentlemen,
Your humble Servant,
D. H. DAVIES.

*No. 27, Carburton-street,*

*Fitzroy-square.*

